# Leveraging Virtual Reality in Pediatric Trauma Education for Pediatric and Emergency Medicine Residents

**DOI:** 10.7759/cureus.113257

**Published:** 2026-07-23

**Authors:** Mariju F Baluyot, Elise Perlman, Dustin A Brinker, Joseph Lopez, Kristin Carmody, Shannon McNamara, Michael Mojica, Joanne Agnant

**Affiliations:** 1 Emergency Medicine, Riley Children's Health, Indiana University Health, Indianapolis, USA; 2 Pediatric Emergency Medicine, Cohen Children's Medical Center, Northwell Health, New Hyde Park, USA; 3 Psychiatry, Northwell Health Physician Partners, New Hyde Park, USA; 4 Trauma, Acute Care Surgery, and Surgical Critical Care, Indiana University Health Ball Memorial Hospital, Muncie, USA; 5 Emergency Medicine, SteadyMD, New York, USA; 6 Emergency Medicine, CityMD, New York, USA; 7 Emergency Medicine, New York University Langone Health, New York, USA

**Keywords:** customized medical education, innovation in medical education, medical education research, medical education technology, medical resident education, pediatric emergency medicine rotation, pediatric trauma, pediatric trauma care, virtual reality simulation, virtual reality (vr)

## Abstract

Objective

Pediatric traumas are high-stakes yet relatively low-frequency events, limiting resident exposure and learning. We developed a low-cost, accessible virtual reality (VR) experience using 360-degree video to supplement pediatric trauma education and explored resident perceptions of its usability, relevance, and educational value.

Methods

We performed a mixed-methods descriptive study at an academic pediatric emergency department. Residents used VR headsets to experience a simulated pediatric blunt abdominal trauma resuscitation which was created with 360-degree video equipment. Interactive access points embedded within the video allowed for review of pediatric-specific considerations and Advanced Trauma Life Support concepts. Pediatric and Emergency Medicine residents provided feedback via anonymous survey with five-point Likert items assessing comfort, ease of use, usefulness, applicability, and interest in curricular integrations. Free-text responses and semi-structured interviews underwent qualitative analysis to extract themes.

Results

Forty-two residents completed the surveys. Most reported the experience was comfortable (n=40, 95.2%) and easy to use (n=42, 100%). Participants rated VR as useful compared with traditional resources (n=42, 100%) and applicable to clinical care (n=41, 97.6%). The majority supported incorporation of VR into the curriculum (n=38, 90.5%) and 69.0% (n=29) would use headsets for independent learning. Qualitative themes highlighted realism, improved appreciation of team dynamics, and the ability to pause for reflection. Learners requested greater interactivity and embedded knowledge checks.

Conclusion

A 360-degree video VR experience was a feasible and positively perceived supplementary educational modality for pediatric trauma training. Learners valued its immersive and flexible format. Our findings reflect learner perceptions rather than objective educational outcomes. Future studies should evaluate effects on knowledge acquisition, behavioral performance, long-term retention, and patient-centered clinical outcomes.

## Introduction

Management of pediatric trauma is an essential skill in Emergency Medicine, especially critical as trauma-related injuries are the leading cause of death in patients aged 1-18 years [1]. Despite this, pediatric trauma cases occur less frequently than in the adult population, limiting resident exposure and opportunities to acquire, practice, and retain essential skills and knowledge [2-4]. Medical simulation has subsequently played a significant role in improving education on pediatric trauma management; however, it requires specialized equipment, space, personnel, and careful coordination of learner and facilitator schedules, often creating logistical barriers to implementation [3,5,6].

Virtual Reality (VR) has been shown to be an innovative and valuable tool in medical education. Like simulation, VR provides learners with an immersive, realistic, and interactive experience to practice a range of technical and non-technical clinical skills. It has been widely used in surgical and procedural training to practice skills without risk of harm to patients, as well as teaching scenarios that are too difficult or costly to replicate, such as mass casualty incidents [7-13]. Additional advantages include asynchronous learning opportunities (removing the need to coordinate learner and facilitator schedules, equipment, and space) and the ability for repeated exposure to rare clinical events.

Despite growing interest in VR-based medical education, evidence supporting its use in pediatric trauma training remains limited. Most VR-based trauma education studies have focused on adult trauma scenarios, while pediatric specific applications remain scarce [14-16]. Recent reviews have highlighted a paucity of evidence examining VR-based pediatric trauma education, particularly among resident learners, and have identified a need for scalable educational approaches that can supplement simulation experiences [17-19].

One challenge to broader adoption of VR in medical education is the cost and complexity associated with accessing fully immersive, interactive, and relevant VR scenarios. Creation of these experiences often requires collaboration with software developers and graphic designers [18,20]. In contrast, 360-degree video offers a more accessible and lower-cost alternative by using a 360-degree camera to capture real clinical scenarios that can be viewed with VR headsets. This approach preserves many of the immersive benefits of VR while requiring fewer technical and financial resources, making it a potentially practical tool for residency education [19,21-25].

However, the use of 360-degree video for pediatric trauma education has received minimal investigation. While it has shown promise in other areas of healthcare education, published studies have largely focused on feasibility, immersion, and learner engagement, with little attention to pediatric trauma or resident-specific training [19,21]. To our knowledge, no published studies have specifically evaluated the use of 360-degree video as an educational modality for pediatric trauma resuscitation among pediatric and emergency medicine resident physicians. Furthermore, a recent systematic review found that most VR education studies evaluate learner reactions and knowledge acquisition, with limited evidence regarding optimal implementation strategies and educational contexts, underscoring the need for early studies examining novel VR approaches in pediatric trauma education [26]. Our objective was to incorporate a 360-degree video pediatric trauma resuscitation experience into resident education and evaluate learner perceptions regarding its usability, educational value, and potential role within pediatric trauma training curricula.

## Materials and methods

Creation, design, and implementation of the VR experience

Through collaboration with Pediatric Emergency Medicine education leadership and a medical education innovations team, the authors designed and created a VR experience utilizing a 360-degree video camera (Insta360 ONE X2; Shenzhen, China) to record a simulated pediatric trauma resuscitation. The video recording featured an interprofessional Pediatric Emergency Medicine resuscitation team (attending physician, fellow, residents, nurses, patient care technicians) managing a simulated pediatric trauma patient (SimJunior mannequin, Laerdal; Stavanger, Norway) in the pediatric trauma bay of the authors’ institution. The primary perspective of the video is from an observer.

The scenario depicted the initial resuscitation of a pediatric patient with blunt abdominal trauma and emphasized both clinical management and non-technical skills, including role clarification and closed-loop communication. Interactive “access points” were embedded throughout the 360-degree video recording using video editing software (FinalCut ProX; Cupertino, California) to allow learners to pause the scenario and access additional supplemental educational content. These modules included the use of equipment specific to pediatric patient care (such as the Broselow tape, airway supplies, intraosseous devices) and Advanced Trauma Life Support (ATLS) concepts such as the primary survey, use of adjunct tests, imaging, and the secondary survey.

**Figure 1:**
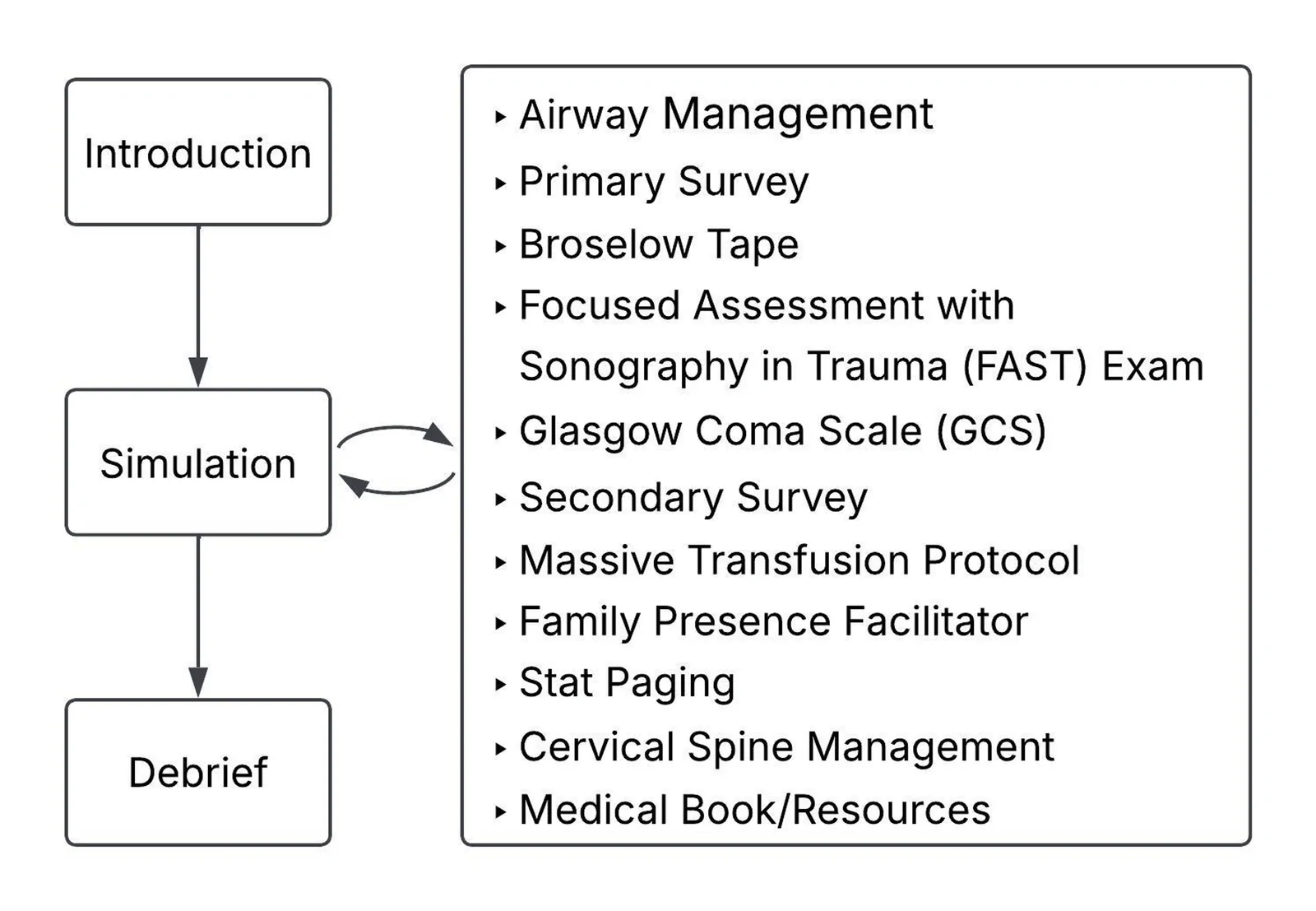
Virtual reality experience content and workflow Image credit: Created by the authors using Lucidchart (Lucid Software Inc., South Jordan, Utah, US).

The complete VR experience required approximately 25 minutes to complete, including a 10-minute immersive simulation and recorded debrief and 15 minutes of supplemental educational content. Learners were able to navigate freely through the simulation and supplemental educational content with ability to pause and replay videos. A workflow diagram of the VR experience is shown in Figure 1.

**Figure 2:**
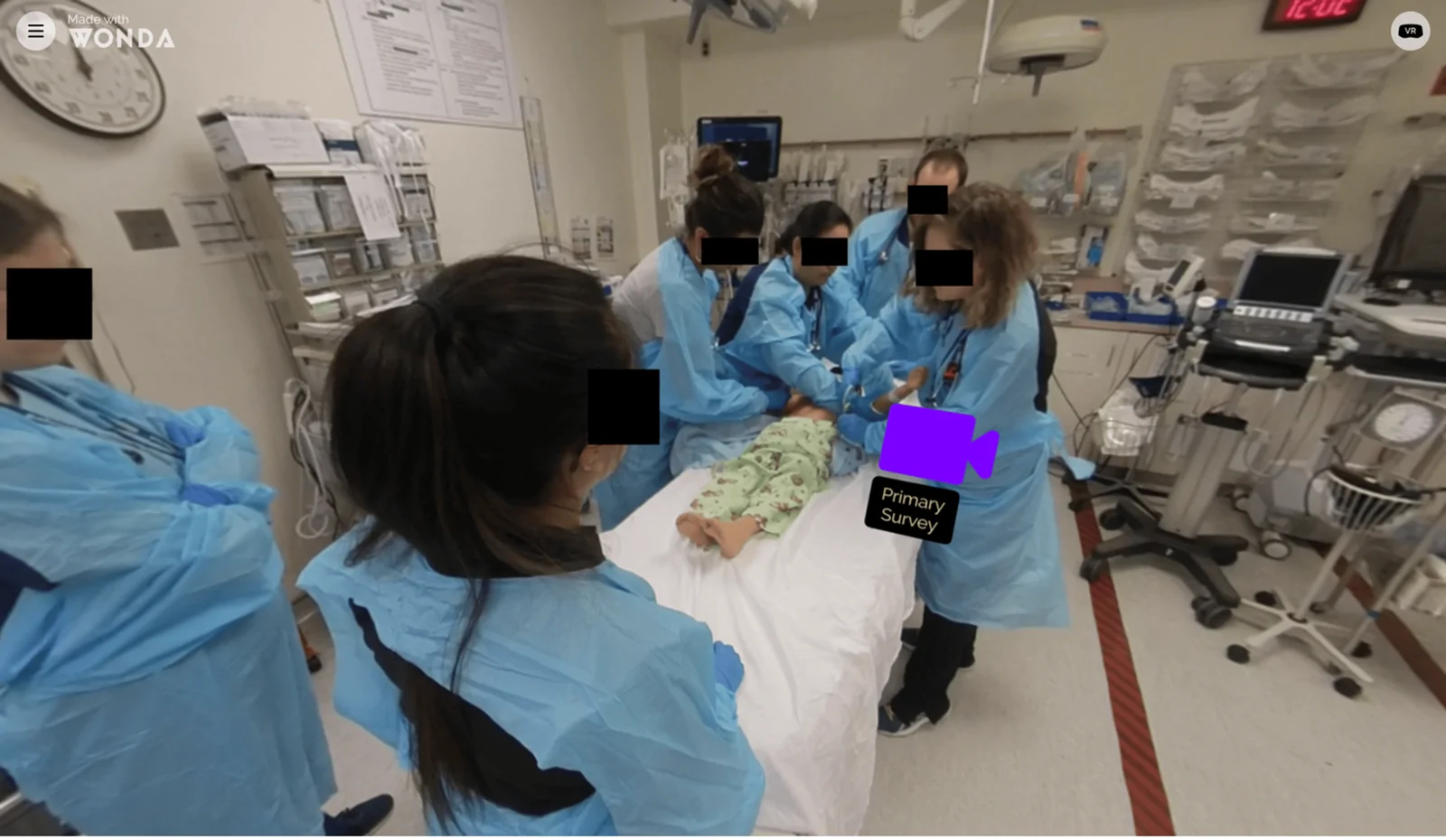
Screenshot of the virtual reality experience

The experience was completed independently without real-time faculty facilitation or debriefing; however, faculty were available for technical support if needed. A representative screenshot of the VR experience demonstrating an embedded “Primary Survey” access point is shown in Figure 2.

Pediatric and EM residents completing their Pediatric Emergency Medicine rotation viewed the experience using Oculus (Meta Quest; Menlo, California) VR headsets. Upon completion of the VR experience, residents completed anonymous evaluations of the education experience.

Study design, setting and population

This was a descriptive mixed-methods study testing a new educational intervention. NYU Langone Health Institutional Review Board's approval was obtained prior to this study (approval no. i19-01009_CR6). The study was conducted at an academic Pediatric Emergency Department and Level 2 Pediatric Trauma Center. Study participants were Pediatric and Emergency Medicine residents at all levels of post-graduate training. Subjects were approached via email for study participation and provided informed consent for the semi-structured interviews after completing the VR experience. Recruitment took place between September 2020 and April 2021.

Feasibility and acceptability outcomes

After completion of the VR experience, learners completed a brief, anonymous questionnaire administered through REDCap (Nashville, Tennessee) to capture their initial impressions of the experience as part of the educational evaluation. The questionnaire was developed by the study team to evaluate this educational intervention and underwent iterative review and refinement by faculty with expertise in Pediatric Emergency Medicine, medical education, and simulation to establish content validity.

The questionnaire evaluated learner preferences for educational modalities and perceptions of the VR experience, including comfort, ease of use, relevance of information, perceived knowledge acquisition, overall satisfaction, and perceived value compared with traditional educational approaches. Participants also provided feedback regarding potential incorporation of the VR experience into residency curricula. At the end of the survey, residents could provide additional free-text comments and critiques.

Study protocol

A study co-investigator (DB) who did not play a role in the evaluation of residents during their Pediatric Emergency Medicine rotations approached potential participants via email after completion of the education and obtained informed consent for the interviews. The authors created, piloted, and revised a semi-structured guide of open-ended questions based on participant feedback (Appendix A). Using this guide, the study co-investigator conducted interviews over an online conference platform (Zoom; San Jose, California) to gather participant perspectives while maintaining their anonymity. Interviews lasted approximately 20-30 minutes. Interview audio was recorded digitally over Zoom and transcribed with a third-party professional service (Transcription Outsources, LLC; Denver, Colorado) for use in data analysis. No personal identifiable information was collected during the audio recording or transcription process.

Data analysis

Quantitative survey data were summarized using descriptive statistics. Responses to the five-point Likert scale items describing comfort, ease of use, usefulness, and applicability were reported as frequencies and percentages. Microsoft Excel (Microsoft Corp., Redmond, WA, USA) was used to generate graphical representations of the results.

Qualitative data from free-text survey responses and semi-structured interview transcripts were analyzed using descriptive thematic analysis. Initial coding was performed independently by the first author (MB), followed by review of the coding framework by two additional investigators (KC and JA) with prior qualitative research experience. Differences in code interpretation were resolved through discussion until consensus was reached. Codes were subsequently grouped into broader themes that reflected participants’ experiences and perceptions of the VR curriculum.

Because only two semi-structured interviews were completed, thematic saturation was not formally assessed. Interview findings were therefore interpreted alongside free-text survey responses to provide additional contextual understanding of learner perceptions rather than as a comprehensive qualitative analysis.

## Results

A total of 42 residents completed the VR experience and post-experience surveys, and two residents participated in the semi-structured interview. Please see Table 1 for full demographics.

**Table 1:** Demographics of the study population

	N (total = 42)	%
Residency Program		
Pediatric	20	47.6
Emergency Medicine	22	52.4
Post-graduate year of training (PGY)		
PGY-1	14	33.3
PGY-2	13	31.0
PGY-3	9	21.4
PGY-4	6	14.3

Learning methods

**Figure 3:**
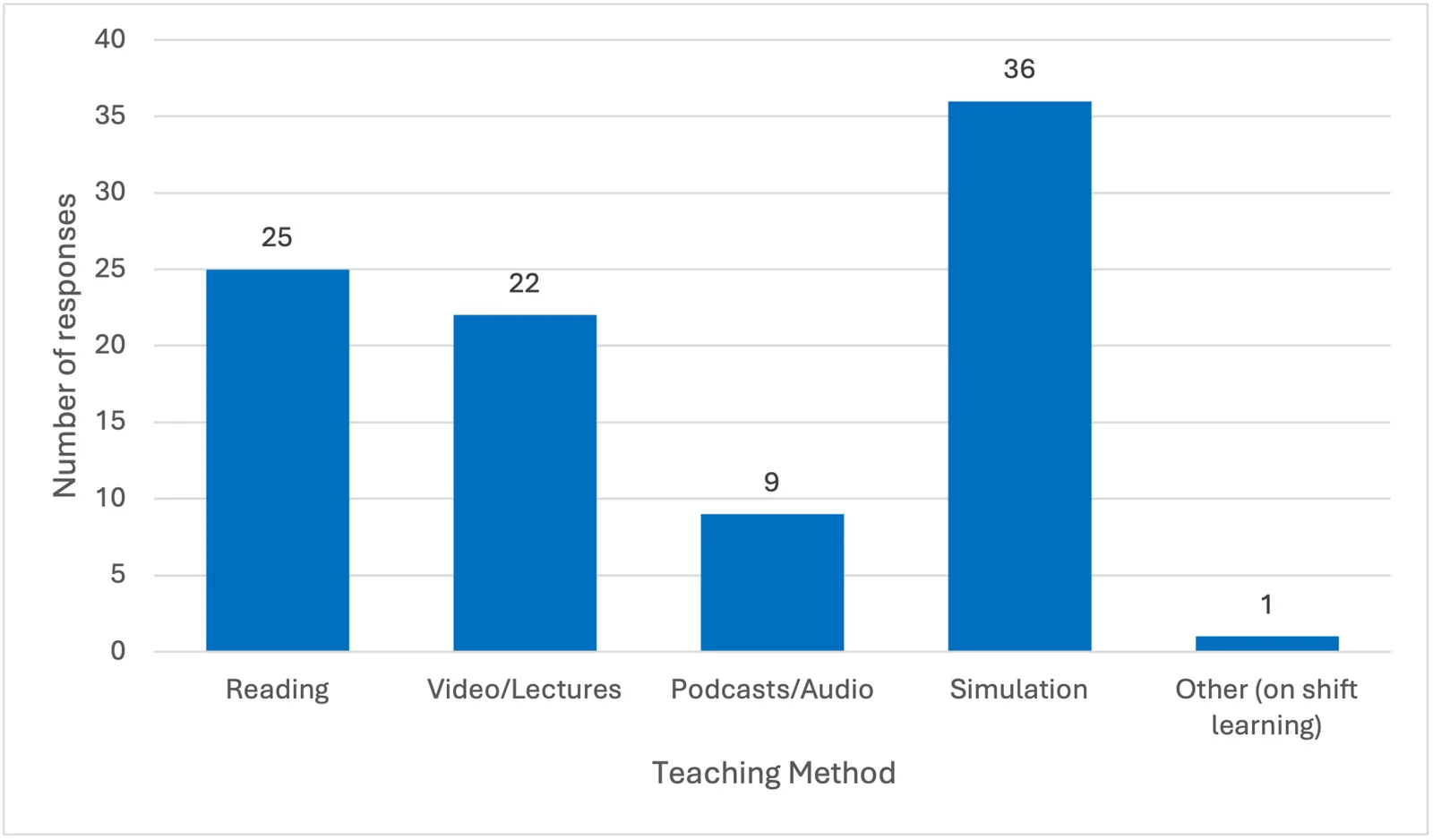
Learner's preferred teaching methods for pediatric trauma

Residents were asked to select their traditional method(s) of learning during the Pediatric Emergency Medicine rotation, as shown in Figure 3.

The majority of residents supplemented their clinical rotation with simulation (n=36, 85.7%), reading (n=25, 59.5%), video lectures (n=22, 52.4%), with fewer residents learning through podcasts/audio (n=9, 21.4%). One resident (n=1, 2.4%) indicated “Other” with their learning style reported as “on shift learning.” 

Curriculum evaluation

**Figure 4:**
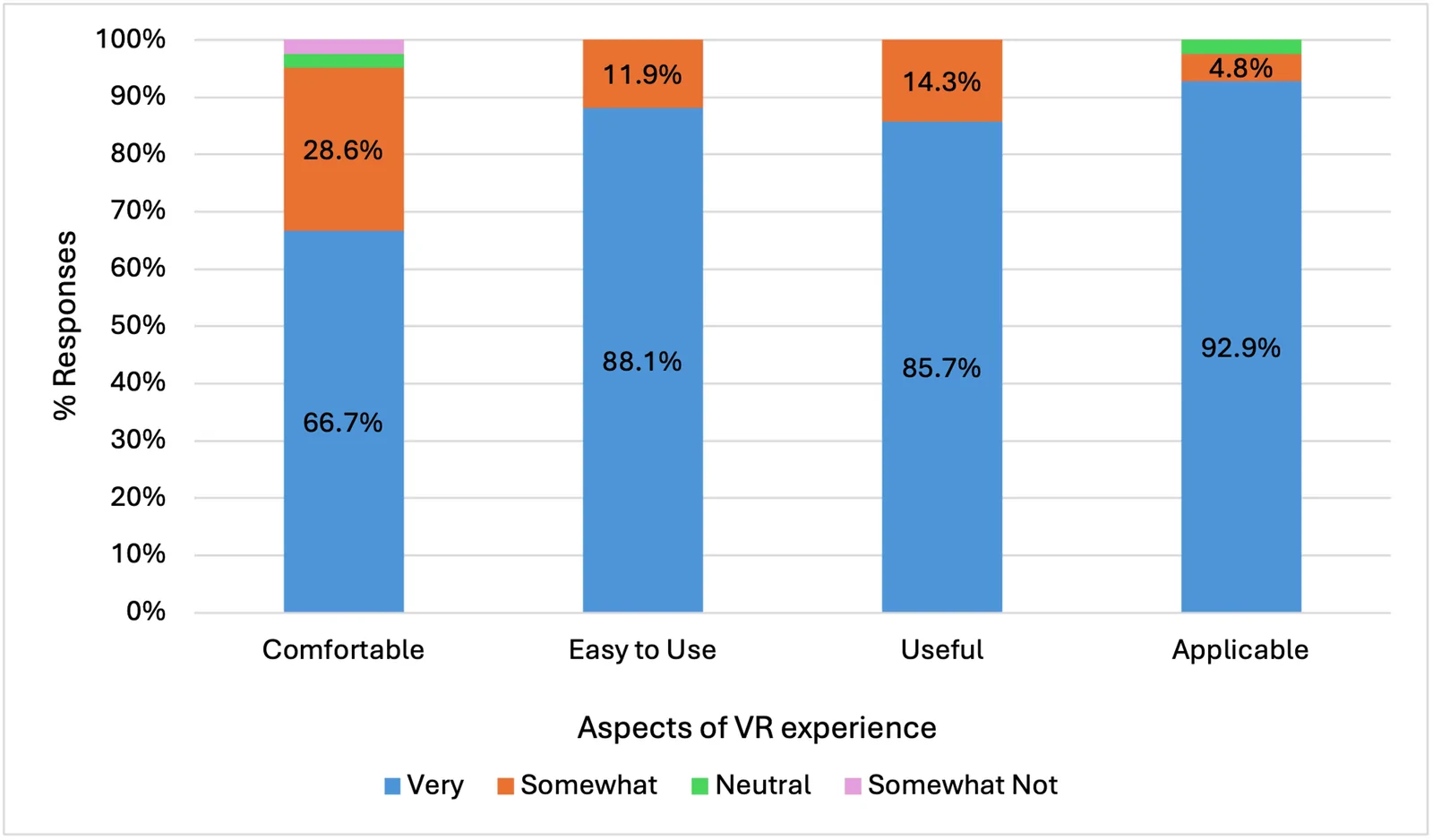
Responses on the virtual reality experience based on comfort, ease of use, usefulness, and applicability

The post-experience survey was used for evaluation of the new curriculum. Questions asked for feedback about comfort of the experience, ease of use, and usefulness of VR as an educational method, and applicability of the information presented (Figure 4).

Comfort

Most participants indicated that they found the VR experience ‘very comfortable’ (n=28, 66.7%) or ‘somewhat comfortable’ (n=12, 28.6%).

Ease of Use

Most learners indicated that they found the VR headset ‘easy to use’ (n=37, 88.1%) or ‘somewhat easy to use’ (n=5, 11.9%).

Usefulness

Most residents indicated that they found the VR experience overall useful when compared to existing resources, such as lectures, podcasts, and simulation, indicating ‘very useful’ (n=36, 85.7%) or ‘somewhat useful’ (n=6, 14.3%).

Applicability

Most participants found the information presented ‘very applicable’ (n=39, 92.9%) or ‘somewhat applicable’ (n=2, 4.8%).

**Figure 5:**
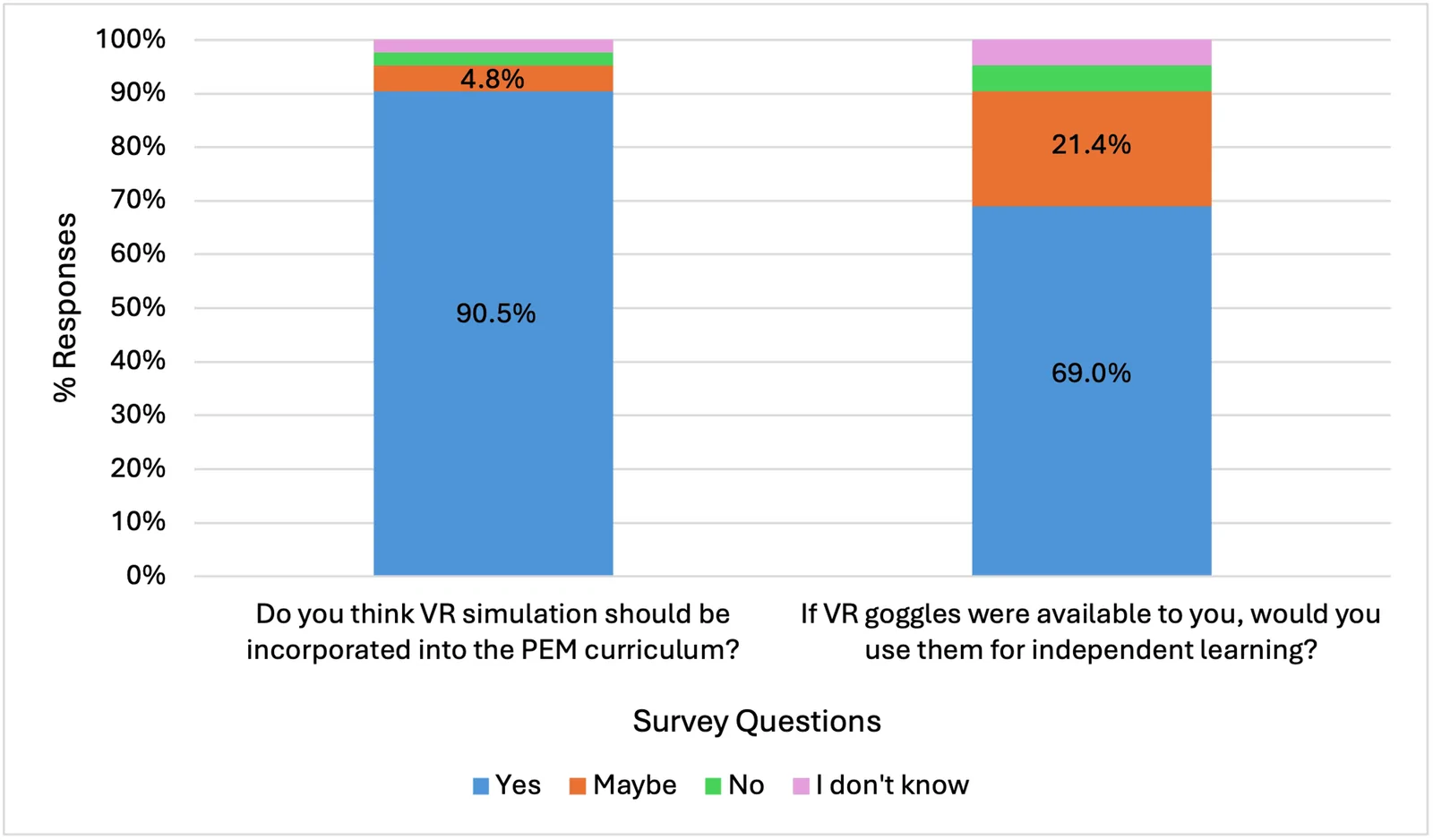
Additional survey responses

Additional questions inquired about the opinions of the participants on incorporation of VR into their regular curriculum and whether they would use VR headsets for independent learning (Figure 5).

Most participants indicated that they thought VR should be incorporated into the Pediatric Emergency Medicine curriculum (n=38, 90.5%). Additionally, most indicated that they would use VR headsets for independent learning if they were available (n=29, 69.0%). 

Survey responses and semi-structured interviews

Qualitative analysis was conducted on transcribed curriculum feedback from study participants as well as two post-experience interviews conducted by the study co-investigator. Themes with select quotations are presented in Table 2. 

**Table 2:** Feedback and interview themes with select quotes

Theme	Subtheme	Selected quotes
Experiential Learning	Realism	"Feel like you’re in a room."
		"Actually feeling like you’re in the room, and seeing the decisions being made in real time."
		"The virtual reality like did a good job at incorporating those things to help prepare you for a real situation like that."
	New Learner	"I had never been in a code before so seeing... it was like giving me a sense of if I ever am in a code, like this is what my role could possibly look like."
		"It would be really good towards quickly dropping someone into a new setting... and then getting them up to speed fairly quickly."
		“It would be great to use in the beginning of the year to get residents acclimated to the trauma slot and the way traumas are run.’
	Learning algorithms	It’s more important to understand the algorithm and when to apply specific principles, and I think that VR does as good as, if not a better job, than traditional lecture style.
Learning adjunct	Pause & Reflect	"You can kind of pause time, think critically, and then continue after you make your educated guess..."
		"In a real scenario... you won’t have the time to stop in the middle of each step and teach."
	Enhance learning	"Virtual reality is definitely more... effective in helping me learn because reading it is much different than actually being in a room."
		"It’s a visual medium... and you can experientially learn a little bit easier through a guided course."
	Patient Safety	“You can’t harm a patient or family if you’re just doing a virtual reality scenario as opposed to doing like an actual scenario where someone’s life is actually at stake.”
		“[We could] do this for real life scenarios and then watch to determine areas for improvement.”
Areas for Improvement	Engagement & Interactivity	"I think having multiple scenarios that run in parallel—like a decision tree."
		“I really liked pushing the buttons to play the videos, maybe you could increase the interaction, like ask a question and I have to choose the answer to move on, and the video discusses it.”
		“Can do questions at the end / multiple choice.”
		“It would have been cool to have an interactive portion, such as some sort of quiz at the end.”
		“Maybe more side videos/ interactive elements. Video is great as it is, but more interaction/ options to answer questions would be great.”
	Tailoring to learner level	“Have different levels of sim for people with different knowledge base and more experience.”
		“Skip the teaching pieces if you are familiar with the topic.”
Other limitations	Virtual vs physical	“A complication that I don’t necessarily think that a VR experience would be able to replicate because it’s a tactile physical exam.”
	Art of medicine	“For the art of medicine maybe the VR component has less utility because there is a definitive end point to each scenario.”

## Discussion

VR is an innovative and valuable teaching method in medical education, offering immersive and asynchronous learning experiences that can supplement traditional education in medical training. This study explored perspectives from pediatric and emergency medicine residents on the implementation of a 360-degree video VR experience for pediatric trauma education. Overall, feedback suggests that learners perceived 360-degree VR as a feasible, engaging, and acceptable educational modality for pediatric trauma training.

Participants reported high levels of comfort, usability, perceived educational value, and enthusiasm for incorporating VR into future residency curricula. Many learners commented positively on the realism of the environment and the ‘observer’ perspective, allowing them to view the resuscitation from a broader perspective and have a better understanding of team dynamics, communication, and workflow. Another notable theme included the ability to ‘pause time,’ which gave participants the opportunity to process and reflect upon teaching points at their own pace. Participants also expressed a desire for increased interactivity during the experience, such as integrating decision-making prompts, embedded quizzes, and branching logic for more advanced learners to enhance engagement and assess knowledge acquisition. Together, the quantitative and qualitative findings suggest that a 360-degree video approach is a feasible and well-accepted method for delivering pediatric trauma education.

These findings are consistent with a growing body of literature demonstrating high learner acceptance of VR across medical education. Previous studies have similarly reported favorable ratings of immersion, engagement, and learner confidence following VR-based trauma education [14,16,27]. Likewise, a recent systematic review concluded that VR consistently demonstrates positive learner reactions comparable to traditional simulation but emphasized that evidence supporting improvements in objective learning outcomes remains limited [26]. Our findings extend this literature by suggesting that a low-cost, 360-degree video approach is both feasible and well-accepted for complex, high-stakes, and relatively low-frequency clinical scenarios such as pediatric trauma education, an area in which published evidence remains scarce.

Although this study did not directly compare 360-degree video with traditional simulation or fully immersive interactive VR, participants perceived several practical advantages of this approach. Compared with custom-built VR applications described in the literature, 360-degree video can be created using commercially available cameras and conventional video-editing software, potentially reducing development costs and technical barriers [19,21]. These characteristics may make 360-degree video particularly attractive for medical training programs seeking to supplement existing simulation curricula with asynchronous educational experiences.

Limitations and future directions

This study has several limitations. First, this was a single-center feasibility study with a relatively small sample size, particularly for the qualitative interviews, limiting transferability of our findings. Because only two interviews were completed, thematic saturation was not achieved, and qualitative findings should be interpreted as illustrative rather than comprehensive. Second, outcomes relied on the self-reported perceptions of the participants, introducing the possibility of response and social desirability bias. There were no objective assessments of knowledge acquisition, procedural skills, teamwork performance, or clinical outcomes. Third, the study did not include a control group or pre-post assessment, precluding conclusions regarding educational effectiveness or comparisons with traditional educational approaches. Lastly, long-term retention, behavioral changes, and clinical implementation were not evaluated, limiting understanding of sustained educational impact of the intervention.

Future work should build upon these feasibility findings by evaluating objective educational outcomes, including knowledge acquisition, behavioral performance during simulated or clinical trauma resuscitations, and long-term knowledge retention. Comparative studies examining 360-degree video alongside traditional simulation and fully immersive interactive VR would help define the optimal role of each modality within pediatric trauma education. In addition, learners consistently expressed interest in greater interactivity, suggesting that future iterations should explore embedded knowledge checks, branching clinical decision pathways, and adaptive learner feedback to enhance engagement and educational effectiveness.

## Conclusions

This study demonstrated that a 360-degree video VR experience was a feasible and positively perceived educational supplement for pediatric and emergency medicine residents within an academic training program. Participants reported high levels of comfort, usability, and perceived educational value. Learners valued the realism and flexibility of the experience and expressed interest in expanding the use of VR to additional clinical scenarios, increased interactive elements, and knowledge assessments. These preliminary findings support the potential role of accessible 360-degree VR as a supplementary educational modality for pediatric trauma training. However, conclusions regarding educational effectiveness cannot be drawn from learner perceptions alone; future studies should evaluate objective measures of knowledge acquisition, behavioral performance, long-term retention, and clinical outcomes to better define the role of VR within pediatric and emergency medicine residency education.

## Appendices

Interview prompts

1)    How did you feel about the VR experience?

2)    How did you feel the VR experience compares to lectures or readings about pediatric trauma?

3)    How did you feel the VR experience compares to simulation?

4)    How did you feel the VR experience compares to a real pediatric trauma?
